# Meta-Analysis Assessment of GP210 and SP100 for the Diagnosis of Primary Biliary Cirrhosis

**DOI:** 10.1371/journal.pone.0101916

**Published:** 2014-07-10

**Authors:** Shi-Ling Hu, Feng-Rong Zhao, Qin Hu, Wei-Xian Chen

**Affiliations:** 1 The Department of Laboratory Medicine, the Second Hospital Affiliated to Chongqing Medical University, Chongqing, China; 2 The Department of Gynecology and obstetrics, Youyang People’s Hospital, Chongqing, China; University of Utah School of Medicine, United States of America

## Abstract

**Purpose:**

To conduct a systematic review of included studies assessing the association of GP210 and SP100 with the risk of primary biliary cirrhosis (PBC) using meta-analysis.

**Methods:**

Five databases, the Cochrane Library, MEDLINE, VIP, CNKI, WANFANG were used to detect the role of GP210 and SP100 in diagnosis of PBC. Approximately 13,000 participants from several countries were included in this analysis. Meta-DiSc statistical software was used for analysis.

**Results:**

25 studies on GP210 and 21 studies on SP100 were included in the meta-analysis. The DOR, sensitivity, specificity of GP210 in diagnosis of PBC were 24.854 (11.957–51.660), 0.272 (0.257–0.288), 0.985 (0.982–0.988), respectively, and they were 9.133 (4.739–17.600), 0.231 (0.213–0.249), 0.977 (0.973–0.981) for SP100.

**Conclusion:**

Our meta-analysis indicated both GP210 and SP100 had high specificity but low sensitivity in diagnosis of PBC.

## Introduction

Primary biliary cirrhosis (PBC) is a chronic, progressive autoimmune disease that is characterized by non-suppurative inflammation of small bile ducts, and the destruction and fibrosis of liver cells, and may progress from cirrhosis to hepatic failure. In addition to liver biochemical tests and histology, the detection of autoantibodies is an essential adjunct for the diagnosis of PBC. According to the diagnosis criteria proposed by the American Association for the Study of Liver Diseases (AASLD), anti-mitochondrial antibodies (AMA) are considered to be the “gold biomarker” for the diagnosis of PBC [1], but people may slip through the net, and the prognostic value of the markers is not widely accepted [2].

Over 60 types of autoantibodies have been detected in PBC patients, some of which have been considered PBC-specific and are utilized as routine PBC diagnostic markers, such as anti-nuclear antibodies (ANAs) [3], which have been recognized as specific targets of PBC. Among these, two subtypes of ANA, one that recognizes nuclear pore membrane protein gp210 (GP210) and another against nuclear body protein sp100 (SP100), have been reported to have a sensitivity of 15–40% [4]–[6] and 20–40% [4], [7], [8], respectively, whereas specificities of both GP210 and SP100 to PBC are greater than 95% [4], [5], [9]. Conversely, while AMAs are not associated with disease progression, ANAs are associated with disease severity and clinical outcome, and are therefore markers of poor prognosis [5], [10]–[13]. The aim of this meta-analysis was to evaluate the diagnostic accuracy of GP210 and SP100 for PBC.

## Methods

### Search Strategy

The literature search was carried out using the Chinese National Knowledge Infrastructure, the Technology of Chongqing, WANFANG data, the Cochrane Library, and MEDLINE databases, without limits on ethnicity or geographic region. The following keywords were used in searching: “GP210 or SP100” or “ANA or anti-nuclear antibodies” and “primary biliary cirrhosis.” Furthermore, to obtain additional relevant articles, we scanned conference summaries and reference lists of retrieved studies, as well as review articles, and even contacted authors to obtain further information, if necessary.

### Eligibility Criteria

Studies were included in the meta-analysis if they met the following criteria: (i) assessed the diagnostic accuracy of the GP210 or SP100 test on PBC in full-text articles; (ii) presented sensitivity and specificity or sufficient information to construct two-by-two tables; (iii) where data sets overlapped or were duplicated, only the largest sample size of patients or the most recent information was included. All identified studies were reviewed independently for eligibility by two investigators. Studies not published in English or Chinese were excluded after identification.

### Data Extraction

Data were extracted independently by two investigators and cross-checked to reach a consensus. The following variables were extracted: name of the first author; year of publication; country where the study was performed; ethnicity of the study population; control sources; PBC diagnosis criteria; antibody testing methods; antibody type; test results, including true positive, false positive, false negative, and true negative; sensitivity and specificity; and essential sample size. The study quality was assessed using the quality assessment of diagnostic accuracy studies (QUADAS) tool. We evaluated each article independently, and discussed discrepancies when they were found.

### Statistical Analysis

Statistical analysis was conducted using Meta-DiSc statistical software, version 1.4 (Unit of Clinical Biostatistics, Ramony Cajal Hospital, Madrid, Spain), and Review Manager 5.2 (Oxford, UK: The Cochran Collaboration). The accuracy indexes of GP210 and SP100, such as diagnostic odds ratio (DOR), sensitivity, specificity, positive likelihood ratio (LR+) and negative likelihood ratio (LR−), were pooled by meta-analysis. The Q test and *I^2^* test were carried out to examine whether variations were caused by heterogeneity or sampling errors (chance). Fixed-effects methods were used if the result of the Q test was not significant (p>0.10 or I^2^<50%), or the random-effects model was used. Subgroup analysis was performed to assess whether threshold effect and heterogeneity existed among studies, according to different measurement methods and geographical regions. The geographical regions used were Europe, South America, and Asia.

Summary receiver operator characteristic (SROC) curves, which show the relationship between sensitivity and 1-specificity, were used for evaluating the global summary of test performance, and the area under the SROC curve represents the overall performance of the detection method. Q* values, which show the point where sensitivity equals specificity, were calculated on the basis of the SROC curves. All p values are two-sided, and p<0.05 was regarded as statistically significant.

## Results

### Literature Search

**Figure 1:**
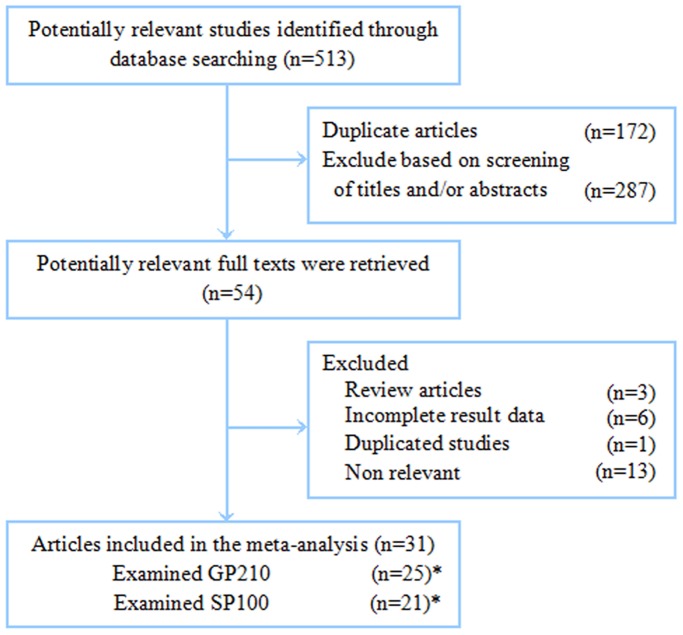
Flowchart of studies included in the meta-analysis.

A flow diagram of our literature search is illustrated in Figure 1. Total searches yielded 513 entries. After the removal of 172 duplicates, 341 titles and abstracts were assessed; 54 articles appeared to be potentially relevant for inclusion in the review. A total of 24 articles were excluded for the following reasons: 13 had no direct link with the main subject; three were reviews; six had incomplete results data, and one duplicated reports from the same study population. The remaining 31 articles [4]–[10], [12]–[35], including 25 [4]–[6], [9]–[10], [12]–[15], [17]–[23], [26], [28]–[35] on GP210 and 21 [4], [6]–[10], [12], [14]–[25], [27], [29] on SP100 (15 [4], [6], [9], [10], [12], [14], [15], [17]–[23], [29] articles reported both GP210 and SP100), were incorporated into the meta-analysis.

### Study Characteristics

The baseline characteristics of all included studies are summarized in Table 1. A total of 25 studies on GP210 (19 studies in English [4], [5], [9], [10], [12], [13], [18]–[21], [26], [28]–[35] and six in Chinese [6], [14], [15], [17], [22], [23]) were published between 1988 and 2013 (Table 1), and involved a total of 2,806 PBC patients and 7,494 controls. Of these 25 studies, 15 were carried out in Asia [5], [6], [9], [12]–[15], [17], [19], [20], [22], [23], [29], [31], [33], five in Europe [10], [28], [30], [32], [35], and five in South America [4], [18], [21], [26], [34]; 56% (14 of 25) using immunoblotting and 48% (12 of 25) with the ELISA method (one article [26] was conducted using two methods). A total of 21 studies on SP100 (13 in English [4], [8], [9], [10], [12], [18]–[21], [24], [25], [27], [29] and eight in Chinese [6], [7], [14]–[17], [22], [23]) were published between 1990 and 2013 (Table 1), and comprised a total of 2,140 cases and 6,976 controls. Of those studies, 13 were conducted in Asia [6], [7], [9], [14]–[17], [19], [20], [22], [23], [29], five were conducted in Europe [8], [10], [24], [25], [27], and three were conducted in South America [4], [18], [21]; 47.6% (10 of 21) using immunoblotting and 52.4% (11 of 21) with the ELISA method.

**Table 1:** Characteristics of studies included in the meta-analysis of the diagnosis of PBC using the GP210 and SP100 test.

Author	Time	Region	Criteria	Methods	gp210				sp100				Scores^&^
					TP	FP	FN	TN	TP	FP	FN	TN	
Du HH (22)	2013	China	AASLD2000	ELISA*	54	26	75	1860	36	15	93	1871	9
Imura-Kumada S (19)	2012	Japan	–	ELISA*	2	1	33	189	11	2	24	188	7
Peng WH (17)	2012	China	AASLD2009	IB#	20	12	28	220	8	2	40	230	9
Saito H (9)	2012	Japan	–	IB#	32	11	48	45	11	2	69	54	8
Xiao H (6)	2012	China	AASLD2000	IB#	19	10	15	286	12	6	22	290	9
Wu J (23)	2012	China	AASLD2000	IB#	49	7	80	1199	34	12	95	1194	9
Lu JX (15)	2011	China	AASLD2009	ELISA*	47	1	60	78	37	2	70	77	9
Hu C (20)	2011	China	AASLD2009	ELISA*	68	5	130	80	51	14	147	71	9
Tang YM (14)	2011	China	AASLD2000	IB#	24	18	47	29	17	17	54	30	8
Stinton LM (18)	2011	Canada	–	IB#	27	0	82	610	27	0	82	610	9
Granito A (25)	2010	Italy	AASLD2009	IB#					30	1	105	156	8
Assassi S (21)	2009	USA	–	ELISA*	1	2	15	799	5	21	11	780	8
Milkiewicz P (4)	2009	Canada	–	ELISA*	32	1	182	66	41	4	173	63	8
Liu Y (16)	2009	China	AASLD2000	IB#					16	4	84	206	9
Gao L (33)	2008	China	AASLD2000	IB#	43	0	97	47					9
Nakamura M (12)	2007	Japan	–	ELISA*	72	0	204	110	24	5	252	105	9
Bauer A (32)	2007	Poland	–	ELISA*	52	2	65	220					8
Nakamura M (5)	2005	Japan	–	ELISA*	23	0	48	192					8
Shao J (7)	2005	China	AASLD2000	IB#					10	1	25	32	9
Bogdanos DP (24)	2003	UK	–	ELISA*					15	8	25	139	7
Muratori P (10)	2003	Italy	–	ELISA*	15	1	81	74	26	17	70	266	8
Miyachi K (31)	2003	Japan	–	IB#	46	0	129	120					10
Kinoshita H (29)	1999	Japan	–	IB#	9	11	28	47	5	7	32	51	8
Itoh S (13)	1998	Japan	–	IB#	25	0	88	162					9
Bandin O (28)	1996	Frence	–	ELISA*	73	2	212	495			285	497	9
Wesierska-Gadek J (30)	1996	Austria	–	IB#	12	0	31	153					8
Sternsdorf T (8)	1995	Germany	–	ELISA*					28	10	39	22	7
Tartakovsky F (26)	1995	USA	–	ELISA*	28	1	138	35					8
Tartakovsky F (26)	1995	USA	–	IB#	27	0	139	36					8
Nickowitz RE (34)	1994	USA		IB#	15	0	144	46					9
Szostecki C (27)	1990	Germany	–	ELISA*					50	9	134	382	8
Lozano F (35)	1988	Spain	–	IB#	16	0	22	277					8

ELISA*: enzyme-linked immunosorbent assay; IB#: immunoblotting; Scores^&^: the No. of items satisfied the 14 standard items from the QUADAS tool.

Abbreviations: AASLD = the American Association for the Study of Liver Diseases.

**Figure 2:**
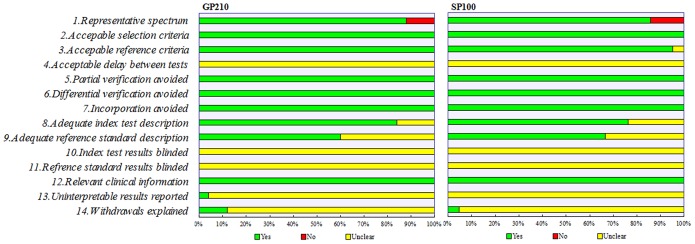
Summary of assessment of the 25 articles on GP210 and 21 articles on SP100 analyzed using the quality assessment of diagnostic accuracy studies (QUADAS) checklist.

All studies included in the meta-analysis were of high quality, with over seven satisfactory items out of 14, as assessed using QUADAS [36]. None of the studies satisfied all criteria of the quality checklist. One study satisfied 10 items of 14 standard items, 14 studies satisfied nine items, 13 studies satisfied eight items and three studies satisfied seven items. Items 4, 10, and 11 (acceptable delay between tests, index test results blinded, and reference standard blinded to index test) of all studies were “unclear”. Studies scored poorly on items regarding the adequate reference standard description, uninterpretable results reported, and withdrawals explained. The included articles’ qualities are shown in Figure 2.

### Meta-analysis

The sensitivities of GP210 ranged from 5.71% to 55.88%, whereas specificities ranged from 61.70% to 100%. Sensitivities and specificities of SP100 ranged from 8.06% to 41.79% and 63.83% to 100%.

**Figure 3:**
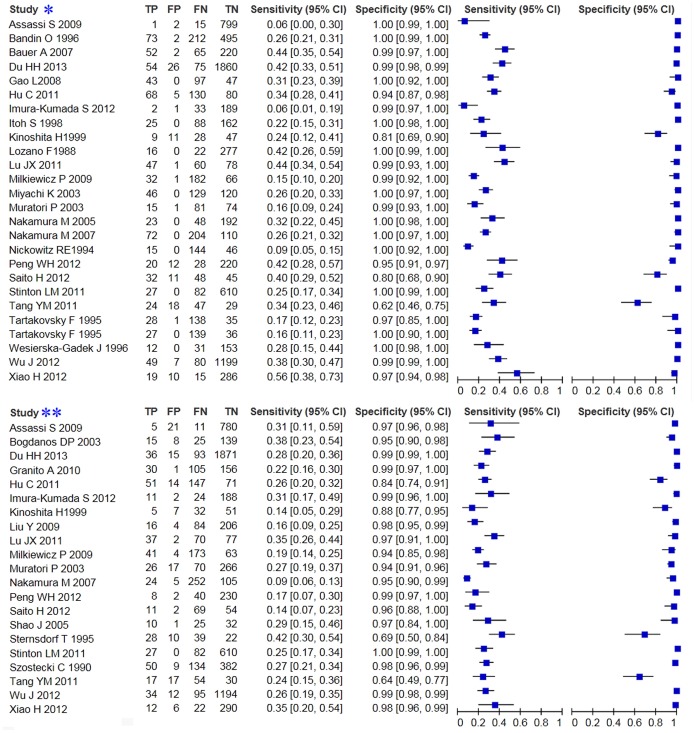
Forest plot of the sensitivity and specificity of GP210 (study*) and SP100 (study**) in the diagnosis of PBC. Only the first author of each study is given. Sensitivity and specificity given with CIs. Abbreviations: CI = confidence interval.

**Figure 4:**
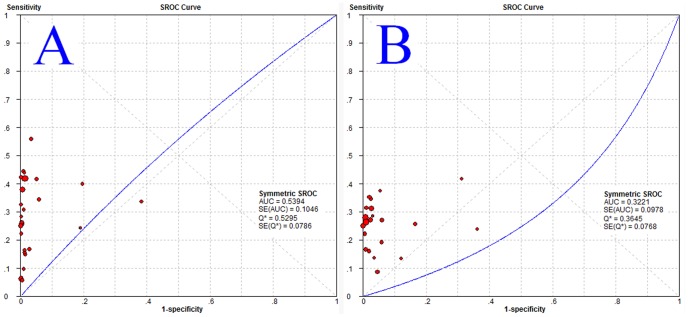
The SROC curve of the GP210 and SP100 test for the diagnosis of PBC. Sample size is indicated by the size of the square. The regression SROC curve indicates the overall diagnostic accuracy. (A) The SROC curves for all data sets about GP210. (B) The SROC curves for all data sets about SP100. Abbreviations: AUC, area under curve; SROC curve, summary receiver operator curve; SE, standard error; Q*, index.

In the pooled analysis for GP210 and SP100, there was significant heterogeneity across studies (p<0.05, I2>50%), so the random-effects model was used to perform the meta-analysis. We analyzed the pooled DOR, sensitivity, specificity, positive likelihood (+LR) and negative likelihood (−LR) of GP210 and SP100. The DOR, sensitivity, specificity, positive likelihood (+LR) and negative likelihood (−LR) of GP210 in the diagnosis of PBC were 24.854 (11.957–51.660), 0.272(0.257–0.288), 0.985 (0.982–0.988), 17.874 (8.706–36.696), and 0.747 (0.696–0.802), respectively, and were 9.133 (4.739–17.600), 0.231 (0.213–0.249), 0.977 (0.973–0.981), 7.205 (3.884–13.366), and 0.805 (0.757–0.855), respectively, for SP100. The forest plots for GP210 and SP100 are shown in Figure 3. The largest area of diagnosis under the summary receiver operator curve for PBC by overall GP210 detection was 0.539, while the SROC was 0.322 for SP100 detection (Figure 4).

### Subgroup Analysis and Publication Bias

The p values of the heterogeneity test for the meta-analysis were all less than 0.1. In consideration that the results might have been influenced by the geographical region and detected method, we performed subgroup analysis according to geographical region and detected method of included studies. The results are listed in Table 2.

**Table 2:** Stratified analyses of the included studies about regions and methods.

type	GP210											SP100									
	Pooled DOR(95%CI)			Pooled SEN(95%CI)		Pooled SPE(95%CI)		pooledLR+		pooledLR−		Pooled DOR(95%CI)		Pooled SEN(95%CI)			Pooled SPE(95%CI)	PooledLR+			PooledLR−
**Regions**																					
European	75.212 (32.024–176.64)		0.290 (0.253–0.329)		0.996 (0.990–0.999)		50.793			0.696	7.935 (3.152–19.978)			0.285 (0.247–0.326)		0.955 (0.941–0.967)		5.905		0.763	
North American	19.164 (6.402–57.364)		0.157 (0.133–0.183)		0.997 (0.994–0.999)		16.236			0.860	19.924 (2.663–149.07)			0.215 (0.173–0.263)		0.983 (0.975–0.989)		14.025		0.793	
Asian	20.899 (8.076–54.085)		0.324 (0.302–0.347)		0.979 (0.974–0.983)		12.784			0.716	8.221 (3.063–22.070)			0.213 (0.191–0.236)		0.980 (0.976–0.984)		6.739		0.825	
China	20.189 (5.653–72.105)		0.379 (0.346–0.412)		0.980 (0.975–0.984)		12.890			0.651	10.866 (3.155–37.425)			0.260 (0.231–0.291)		0.982 (0.978–0.986)		8.668		0.795	
Japan	18.246 (3.342–99.620)		0.266 (0.235–0.298)		0.974 (0.961–0.984)		14.167			0.788	4.201 (1.008–17.502)			0.119 (0.090–0.154)		0.961 (0.938–0.978)		3.654		0.899	
**Methods**																					
ELISA	30.862 (16.223–58.709)	0.273 (0.252–0.295)			0.990 (0.987–0.993)		21.557		0.748		8.049 (3.645–17.777)		0.238 (0.215–0.261)		0.974 (0.968–0.978)			6.183	0.779		
IB	22.002 (7.000–69.152)	0.271 (0.248–0.296)			0.979 (0.974–0.984)		15.259		0.745		11.259 (3.290–38.527)		0.219 (0.190–0.249)		0.982 (0.977–0.987)			9.313	0.827		

Abbreviations: DOR = diagnostic odds ratio, CI = confidence interval, SEN = sensitivity, SPE = specificity, LR+/− = positive/negative likelihood ratio, ELISA = enzyme-linked immunosorbent assay, IB = immunoblotting.

Little difference was found in the sensitivity of GP210 in our stratified analyses of regions. The sensitivity was highest in Asians (32.4%), followed by Europeans (29%), and then in North Americans (15.7%). Different geographical area and selection of cases according to genetic and environmental factors might explain the discrepancy.

**Figure 5:**
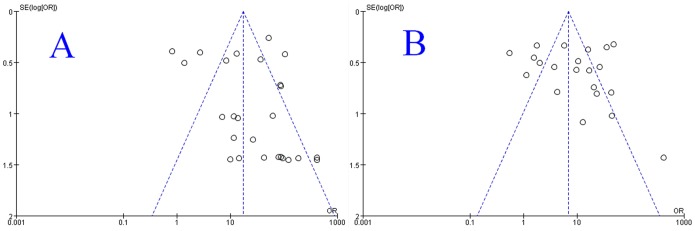
Funnel plot of all included studies. (A) Funnel plot of all included studies for GP210. (B) Funnel plot of all included studies for SP100.

Furthermore, we conducted funnel plots (Figure 5), which demonstrated a somewhat asymmetric curve. This can be explained by the limited number of included studies to assess the publication bias in the meta-analysis.

## Discussion

ANAs are often tested in the manner of a screening tool in patients with suspected systemic lupus erythematosus, progressive systemic sclerosis, or other connective tissue diseases [37]–[40]. In the 1950s, ANAs in PBC were first detected by indirect immunofluorescence on frozen liver tissue sections or cultured cell lines [41]. A positive rate of ANAs has been found in approximately 30–50% of unselected PBC patients [2], [42], [43]. Although the histopathological changes act as the “gold standard” for the diagnosis of PBC, these changes are not observed in all PBC patients. Moreover, the liver is not affected symmetrically, and a single biopsy might simultaneously demonstrate the presence of all histologic stages [44], so many patients are unwilling to accept this examination. AMA is a useful marker for PBC, and has been reported to be present in 80%–96.5% of patients with PBC [45]–[48]. In addition, a meta-analysis showed that the pooled sensitivity and specificity of AMA in the diagnosis of PBC is 84.5% and 97.8%, respectively [49]. Milkiewicz et al. showed that in chronic, presumed autoimmune cholestatic liver disease, both GP210 and SP100 are highly specific for PBC, and might also have been detected in some PBC patients who were negative for conventional AMA antibodies [4]. We conducted a survey regarding both AMA and GP210/SP100, which were detected simultaneously in some of the included studies, and this is summarized in Table 3. Compared with AMA alone to detect PBC, there was a significantly improved sensitivity when AMA was combined with GP210 or SP100.

**Table 3:** The sensitivities of combined AMA with GP210 or SP100 in PBC patients and the positive rate of GP210 or SP100 in AMA-negtive PBC patients.

	*SEN*					positive rate*	
	AMA	GP210	SP100	AMA+SP100	AMA+GP210	GP210	SP100
Milkiewicz P (4)	73.36%	14.95%	19.16%	78.50%	77.57%	10.53%	10.53%
Xiao H (6)	85.29%	55.88%	35.29%	94.12%	94.12%	60.00%	60.00%
Saito H (9)	65.00%	40.00%	13.75%	82.50%	86.25%	33.33%	0
Hu C (20)	92.42%	34.34%	25.76%	92.93%	93.94%	20.00%	6.67%
Muratori P (10)	86.46%	15.63%	27.08%	–	–	15.38%	38.46%
Wu J (23)	65.12%	37.98%	26.36%	–	–	57.78%	60.00%

Abbreviations: SEN = sensitivity.

positive rate*: the positive rate of GP210 or SP100 in AMA-negtive PBC patients.

GP210, which is a type I integral membrane protein that anchors nuclear pore complexes to the pore membrane [50], [51], had a pooled sensitivity of approximately 27%, and a pooled specificity of greater than 97% for PBC in our study. Furthermore, the average of the positive rate of GP210 in AMA-negative PBC patients was 32.84% (10.53–60.00%) (Table 3). Autoantibody to GP210 has been shown to correlate with disease stage, and has been reported as being associated with a worse outcome and likely a more rapid progression of PBC [4], [5], [12], [13]. Milkiewicz et al. detected GP210 in 37% of patients with PBC who had a known bad outcome (progressed sufficiently to require a liver transplant or who died), compared with 10% in a cohort of patients whose outcome had yet to be defined [4].

SP100 has also been detected in AMA-negative PBC patients, and is considered to be a useful marker for serological diagnosis of PBC [42]. Moreover, SP100 positivity is more common in patients with advanced stages of disease, and faster disease progression rates have been observed among SP100-positive PBC patients [52], [53]. In the current study, the pooled sensitivity and specificity of SP100 for PBC was 23.1% and 97.7%, respectively, while the positive rate in AMA-negative PBC patients was 0–60% (Table 3). There were no significant differences in pooled specificity across the strata of geographical regions and measurement methods.

It might be generally difficult to avoid publication bias in a meta-analysis. All of the articles included in our meta-analysis were published papers; nevertheless, numerous unfavorable results may have been found and not published. Therefore, we tried our best to incorporate a higher number of studies, such as papers presented in conferences, to reduce the bias. In addition, heterogeneity is a potential problem when interpreting the consequences in a meta-analysis. However, the Spearman correlation coefficient of sensitivity and 1-specificity was 0.500 (p = 0.391, 0.05), indicating no heterogeneity from threshold effects. Moreover, the pooled DOR of each study was not distributed along a straight line, with the pooled DOR in the forest plots and the Q* (199.67, P = 0.0000) implying a non-threshold effect in the analysis. Diverse geographical regions or measurement methods may contribute to heterogeneity sources. Therefore, we performed the meta-regression analysis and subgroup analysis.

In conclusion, GP210 and SP100 are serviceable as diagnostic markers, especially in patients with PBC negative for AMA. An ideal biomarker should have superior sensitivity and specificity, and for GP210 and SP100 this is insufficient. However, determining PBC diagnoses and prognoses remain challenging. AMA is highly sensitive, and GP210 and SP100 are extremely specific for PBC. It is necessary to combine AMA and the GP210 and/or SP100 antibodies to decrease the misdiagnosis rate in clinical applications.

## Supporting Information

Table S1 **Specific scores of all included studies according to the QUADAS tool.**
(DOC)

Table S2 **The excluded studies and the reasons for exclusion.**
(DOC)

Checklist S1 **Preferred items reporting for the meta-analysis.**
(DOC)
