# Expression of concern: Meta-analysis assessment of GP210 and SP100 for the diagnosis of primary biliary cirrhosis

**DOI:** 10.1371/journal.pone.0348620

**Published:** 2026-05-05

**Authors:** The PLOS One Editors

The *PLOS One* Editors issue this Expression of Concern for this article [1] because of concerns identified about the peer review process. Readers are advised to interpret the article [1] with caution.

The *PLOS One* Editors also wish to make readers aware that there is substantial overlap between the content of this article and that of another article subsequently published in the *Chinese Journal of Hepatology* [2].

An internal editorial assessment has established that this constitutes redundant publication. As the first of the two publications, the *PLOS One* article stands. The *Chinese Journal of Hepatology* has been informed.
