# Supplementary material for: Meta-Analysis Assessment of GP210 and SP100 for the Diagnosis of Primary Biliary Cirrhosis
Source: PLoS One. 2014 Jul 10;9(7):e101916. doi: 10.1371/journal.pone.0101916 (PMC4092088; doi:10.1371/journal.pone.0101916)
Supplement: Table S1 — Specific scores of all included studies according to the QUADAS tool. (DOC) [file pone.0101916.s001.doc]

| GP210 | 4 | 5 | 6 | 9 | 10 | 12 | 13 | 14 | 15 | 17 | 18 | 19 | 20 | 21 | 22 | 23 | 26 | 28 | 29 | 30 | 31 | 32 | 33 | 34 | 35 |
| --- | --- | --- | --- | --- | --- | --- | --- | --- | --- | --- | --- | --- | --- | --- | --- | --- | --- | --- | --- | --- | --- | --- | --- | --- | --- |
| 1.Representative spectrum | **Y** | **Y** | **Y** | **N** | **Y** | **Y** | **Y** | **Y** | **Y** | **Y** | **Y** | **N** | **Y** | **N** | **Y** | **Y** | **Y** | **Y** | **Y** | **Y** | **Y** | **Y** | **Y** | **Y** | **Y** |
| 2.Accepable selection criteria | **Y** | **Y** | **Y** | **Y** | **Y** | **Y** | **Y** | **Y** | **Y** | **Y** | **Y** | **Y** | **Y** | **Y** | **Y** | **Y** | **Y** | **Y** | **Y** | **Y** | **Y** | **Y** | **Y** | **Y** | **Y** |
| 3.Accepable reference criteria | **Y** | **Y** | **Y** | **Y** | **Y** | **Y** | **Y** | **Y** | **Y** | **Y** | **Y** | **Y** | **Y** | **Y** | **Y** | **Y** | **Y** | **Y** | **Y** | **Y** | **Y** | **Y** | **Y** | **Y** | **Y** |
| 4.Acceptable delaY between tests | **U** | **U** | **U** | **U** | **U** | **U** | **Y** | **U** | **U** | **U** | **U** | **U** | **U** | **U** | **U** | **U** | **U** | **U** | **U** | **U** | **U** | **U** | **U** | **U** | **U** |
| 5.Partial verification avoided | **Y** | **Y** | **Y** | **Y** | **Y** | **Y** | **Y** | **Y** | **Y** | **Y** | **Y** | **Y** | **Y** | **Y** | **Y** | **Y** | **Y** | **Y** | **Y** | **Y** | **Y** | **Y** | **Y** | **Y** | **Y** |
| 6.Differential verification avoided | **Y** | **Y** | **Y** | **Y** | **Y** | **Y** | **Y** | **Y** | **Y** | **Y** | **Y** | **Y** | **Y** | **Y** | **Y** | **Y** | **Y** | **Y** | **Y** | **Y** | **Y** | **Y** | **Y** | **Y** | **Y** |
| 7.Incorporation avoided | **Y** | **Y** | **Y** | **Y** | **Y** | **Y** | **Y** | **Y** | **Y** | **Y** | **Y** | **Y** | **Y** | **Y** | **Y** | **Y** | **Y** | **Y** | **Y** | **Y** | **Y** | **Y** | **Y** | **Y** | **Y** |
| 8.Adequate index test description | **Y** | **U** | **Y** | **Y** | **Y** | **Y** | **Y** | **U** | **Y** | **Y** | **Y** | **Y** | **Y** | **Y** | **Y** | **Y** | **Y** | **Y** | **U** | **Y** | **Y** | **Y** | **U** | **Y** | **Y** |
| 9.Adequate reference standard description | **U** | **Y** | **Y** | **Y** | **U** | **U** | **U** | **Y** | **Y** | **Y** | **Y** | **U** | **Y** | **U** | **Y** | **Y** | **U** | **Y** | **Y** | **U** | **Y** | **U** | **Y** | **Y** | **U** |
| 10.Index test results blinded | **U** | **U** | **U** | **U** | **U** | **U** | **U** | **U** | **U** | **U** | **U** | **U** | **U** | **U** | **U** | **U** | **U** | **U** | **U** | **U** | **U** | **U** | **U** | **U** | **U** |
| 11.Refrence standard results blinded | **U** | **U** | **U** | **U** | **U** | **U** | **U** | **U** | **U** | **U** | **U** | **U** | **U** | **U** | **U** | **U** | **U** | **U** | **U** | **U** | **U** | **U** | **U** | **U** | **U** |
| 12.Relevant clinical information | **Y** | **Y** | **Y** | **Y** | **Y** | **Y** | **Y** | **Y** | **Y** | **Y** | **Y** | **Y** | **Y** | **Y** | **Y** | **Y** | **Y** | **Y** | **Y** | **Y** | **Y** | **Y** | **Y** | **Y** | **Y** |
| 13.Uninterpretable results reported | **U** | **U** | **U** | **U** | **U** | **U** | **U** | **U** | **U** | **U** | **U** | **U** | **U** | **U** | **U** | **U** | **U** | **U** | **U** | **U** | **Y** | **U** | **U** | **U** | **U** |
| 14.Withdrawals explained | **U** | **U** | **U** | **U** | **U** | **Y** | **U** | **U** | **U** | **U** | **U** | **U** | **U** | **Y** | **U** | **U** | **U** | **U** | **U** | **U** | **U** | **U** | **Y** | **U** | **U** |

| SP100 | 4 | 6 | 7 | 8 | 9 | 10 | 12 | 14 | 15 | 16 | 17 | 18 | 19 | 20 | 21 | 22 | 23 | 24 | 25 | 27 | 29 |
| --- | --- | --- | --- | --- | --- | --- | --- | --- | --- | --- | --- | --- | --- | --- | --- | --- | --- | --- | --- | --- | --- |
| 1.Representative spectrum | **Y** | **Y** | **Y** | **Y** | **N** | **Y** | **Y** | **Y** | **Y** | **Y** | **Y** | **Y** | **N** | **Y** | **N** | **Y** | **Y** | **Y** | **Y** | **Y** | **Y** |
| 2.Accepable selection criteria | **Y** | **Y** | **Y** | **Y** | **Y** | **Y** | **Y** | **Y** | **Y** | **Y** | **Y** | **Y** | **Y** | **Y** | **Y** | **Y** | **Y** | **Y** | **Y** | **Y** | **Y** |
| 3.Accepable reference criteria | **Y** | **Y** | **Y** | **U** | **Y** | **Y** | **Y** | **Y** | **Y** | **Y** | **Y** | **Y** | **Y** | **Y** | **Y** | **Y** | **Y** | **Y** | **Y** | **Y** | **Y** |
| 4.Acceptable delaY between tests | **U** | **U** | **U** | **U** | **U** | **U** | **U** | **U** | **U** | **U** | **U** | **U** | **U** | **U** | **U** | **U** | **U** | **U** | **U** | **U** | **U** |
| 5.Partial verification avoided | **Y** | **Y** | **Y** | **Y** | **Y** | **Y** | **Y** | **Y** | **Y** | **Y** | **Y** | **Y** | **Y** | **Y** | **Y** | **Y** | **Y** | **Y** | **Y** | **Y** | **Y** |
| 6.Differential verification avoided | **Y** | **Y** | **Y** | **Y** | **Y** | **Y** | **Y** | **Y** | **Y** | **Y** | **Y** | **Y** | **Y** | **Y** | **Y** | **Y** | **Y** | **Y** | **Y** | **Y** | **Y** |
| 7.Incorporation avoided | **Y** | **Y** | **Y** | **Y** | **Y** | **Y** | **Y** | **Y** | **Y** | **Y** | **Y** | **Y** | **Y** | **Y** | **Y** | **Y** | **Y** | **Y** | **Y** | **Y** | **Y** |
| 8.Adequate index test description | **Y** | **Y** | **Y** | **Y** | **Y** | **Y** | **U** | **U** | **Y** | **Y** | **Y** | **Y** | **Y** | **Y** | **Y** | **Y** | **Y** | **U** | **U** | **Y** | **U** |
| 9.Adequate reference standard description | **U** | **Y** | **Y** | **U** | **Y** | **U** | **Y** | **Y** | **Y** | **Y** | **Y** | **Y** | **U** | **Y** | **U** | **Y** | **Y** | **U** | **Y** | **U** | **Y** |
| 10.Index test results blinded | **U** | **U** | **U** | **U** | **U** | **U** | **U** | **U** | **U** | **U** | **U** | **U** | **U** | **U** | **U** | **U** | **U** | **U** | **U** | **U** | **U** |
| 11.Refrence standard results blinded | **U** | **U** | **U** | **U** | **U** | **U** | **U** | **U** | **U** | **U** | **U** | **U** | **U** | **U** | **U** | **U** | **U** | **U** | **U** | **U** | **U** |
| 12.Relevant clinical information | **Y** | **Y** | **Y** | **Y** | **Y** | **Y** | **Y** | **Y** | **Y** | **Y** | **Y** | **Y** | **Y** | **Y** | **Y** | **Y** | **Y** | **Y** | **Y** | **Y** | **Y** |
| 13.Uninterpretable results reported | **U** | **U** | **U** | **U** | **U** | **U** | **U** | **U** | **U** | **U** | **U** | **U** | **U** | **U** | **U** | **U** | **U** | **U** | **U** | **U** | **U** |
| 14.Withdrawals explained | **U** | **U** | **U** | **U** | **U** | **U** | **U** | **U** | **U** | **U** | **U** | **U** | **U** | **U** | **Y** | **U** | **U** | **U** | **U** | **U** | **U** |
