# Supplementary material for: Meta-Analysis Assessment of GP210 and SP100 for the Diagnosis of Primary Biliary Cirrhosis
Source: PLoS One. 2014 Jul 10;9(7):e101916. doi: 10.1371/journal.pone.0101916 (PMC4092088; doi:10.1371/journal.pone.0101916)
Supplement: Table S2 — The excluded studies and the reasons for exclusion. (DOC) [file pone.0101916.s002.doc]

| **Duplicated study：** | Muratori P, Muratori L, Cassani F, Terlizzi P, Lenzi M, et al. (2002) Anti-multiple nuclear dots (anti-MND) and anti-SP100 antibodies in hepatic and rheumatological disorders. Clin Exp Immunol 127:172-175. [PubMed, PMID: 11882049] |
| --- | --- |
| **Review articles：** | 1. Hu CJ, Zhang FC, Li YZ, Zhang X (2010) Primary biliary cirrhosis: What do autoantibodies tell us? World J Gastroenterol 16:3616-3129. [PubMed, PMID: 20677333] 2. Worman HJ (2007) Nuclear envelope protein autoantigens in primary biliary cirrhosis. Hepatol Res 37:S406-411. [PubMed, PMID:17931195] 3. Granito A, Muratori P, Quarneti C, Pappas G, Cicola R, et al. (2012) Antinuclear antibodies as ancillary markers in primary biliary cirrhosis. Expert Rev Mol Diagn 12:65-74. [PubMed, PMID: 22133120] |
| **Incomplete result data:** | 1. Mytilinaiou MG, Meyer W, Scheper T, Rigopoulou EI, Probst C, et al. (2012) Diagnostic and clinical utility of antibodies against the nuclear body promyelocytic leukaemia and Sp100 antigens in patients with primary biliary cirrhosis. Clin Chim Acta 413(15-16):1211-1216. [PubMed, PMID: 22503841] 2. Tsangaridou E, Polioudaki H, Sfakianaki R, Samiotaki M, Tzardi M, et al. (2010) Differential detection of nuclear envelope autoantibodies in primary biliary cirrhosis using routine and alternative methods. BMC Gastroenterol 10:28. [PubMed, PMID: 20205958] 3. Liu H, Liu Y, Wang L, Xu D, Lin B, et al. (2010) Prevalence of primary biliary cirrhosis in adults referring hospital for annual health check-up in Southern China. BMC Gastroenterol 10:100. [PubMed, PMID: 20815889] 4. Miyachi K, Shibata M, Onozuka Y, Kikuchi F, Imai N, et al. (1996) Primary biliary cirrhosis sera recognize not only gp210 but also proteins of the p62 complex bearing N-acetylglucosamine residues from rat liver nuclear envelope. Mol Biol Rep 23(3-4):227-234. [PubMed, PMID: 9112233] 5. Muratori L, Granito A, Muratori P, Pappas G, Bianchi FB (2008) Antimitochondrial Antibodies and Other Antibodies in Primary Biliary Cirrhosis: Diagnostic and Prognostic Value. Clin Liver Dis 12:261-276. [PubMed, PMID: 18456179] 6. Worman HJ, Courvalin JC (2003) Antinuclear antibodies specific for primary biliary cirrhosis. Autoimmun Rev 2:211–217. [PubMed, PMID: 12848948] |
| **Non relevant:** | 1. Luettig B, Boeker KH, Schoessler W, Will H, Loges S, et al. (1998) The antinuclear autoantibodies Sp100 and gp210 persist after orthotopic liver transplantation in patients with primary biliary cirrhosis. J Hepatol 28(5):824-828. [PubMed, PMID: 9625318] 2. Rigopoulou EI, Dalekos GN (2008) Molecular diagnostics of primary biliary cirrhosis. Expert Opin Med Diagn 2(6):621-634. [PubMed, PMID: 23495774] 3. Kempinska-Podhorodecka A, Shums Z, Wasilewicz M, Wunsch E, Milkiewicz M, et al. (2012) TRAF1 Gene Polymorphism Correlates with the Titre of Gp210 Antibody in Patients with Primary Biliary Cirrhosis. Clin Dev Immunol 2012:487521. [PubMed, PMID: 23125866] 4. Ikuno N, Scealy M, Davies JM, Whittingham SF, Omagari K, et al. (2001) A Comparative Study of Antibody Expressions in Primary Biliary Cirrhosis and Autoimmune Cholangitis Using Phage Display. Hepatology 34(3):478-486. [PubMed, PMID: 11526532] 5. Szostecki C, Will H, Netter HJ, Guldner HH (1992) Autoantibodies to the Nuclear Spl00 Protein in Primary Biliary Cirrhosis and Associated Diseases: Epitope Specificity and Immunoglobulin Class Distribution. Scand J Immunol 36(4):555-564. [PubMed, PMID:1384113] 6. Manuel Lucena J, Montes Cano M, Luis Caro J, Respaldiza N, Alvarez A, et al. (2007) Comparison of Two ELISA Assays for Anti-Sp100 Determination. Ann N Y Acad Sci 1109:203-211. [PubMed, PMID:17785307] 7. Nakamura M, Takii Y, Ito M, Komori A, Yokoyama T, et al. (2006) Increased expression of nuclear envelope gp210 antigen in small bile ducts in primary biliary cirrhosis. J Autoimmun 26(2):138-145. [PubMed, PMID:16337775] 8. Nakamura M, Komori A, Ito M, Kondo H, Aiba Y, et al. (2007) Predictive role of anti-gp210 and anticentromere antibodies in long-term outcome of primary biliary cirrhosis. Hepatol Res 37:S412-419. [PubMed, PMID:17931196] 9. Cavazzana I, Ceribelli A, Taraborelli M, Fredi M, Norman G, et al. (2011) Primary Biliary Cirrhosis-related Autoantibodies in a Large Cohort of Italian Patients with Systemic Sclerosis. J Rheumatol 38(10):2180-2185. [PubMed, PMID:21921093] 10. Zografos TA, Gatselis N, Zachou K, Liaskos C, Gabeta S, et al. (2012) Primary biliary cirrhosis-specific autoantibodies in first degree relatives of Greek primary biliary cirrhosis patients. World J Gastroenterol 18(34):4721-4728. [PubMed, PMID:23002341] 11. Invernizzi P, Podda M, Battezzati PM, Crosignani A, Zuin M, et al. (2001) Autoantibodies against nuclear pore complexes are associated with more active and severe liver disease in primary biliary cirrhosis. J Hepatol 34(3):366-372. [PubMed, PMID:11322196 ] 12. Wichmann I, Montes-Cano MA, Respaldiza N, Alvarez A, Walter K, et al. (2003) Clinical Significance of Anti-Multiple Nuclear Dots/Sp100 Autoantibodies. Scand J Gastroenterol 38(9):996-999. [PubMed, PMID:14531539] 13. Nesher G, Margalit R, Ashkenazi YJ (2001) Anti-nuclear envelope antibodies: Clinical associations. Semin Arthritis Rheum 30(5):313-320. [PubMed, PMID:11303304] |
